# Diarrhea Outbreak during U.S. Military Training in El Salvador

**DOI:** 10.1371/journal.pone.0040404

**Published:** 2012-07-18

**Authors:** Matthew R. Kasper, Andres G. Lescano, Carmen Lucas, Duncan Gilles, Brian J. Biese, Gary Stolovitz, Erik J. Reaves

**Affiliations:** 1 U.S. Naval Medical Research Unit 6, Lima, Peru; 2 Madigan Healthcare System, Tacoma, Washington, United States of America; 3 452nd Combat Support Hospital, U.S. Army Reserve, Milwaukee, Wisconsin, United States of America; 4 Universidad Peruana Cayetano Heredia, Lima, Peru; The Australian National University, Australia

## Abstract

Infectious diarrhea remains a major risk to deployed military units worldwide in addition to their impact on travelers and populations living in the developing world. This report describes an outbreak of diarrheal illness in the U.S. military’s 130^th^ Maneuver Enhancement Brigade deployed in San Vicente, El Salvador during a training and humanitarian assistance mission. An outbreak investigation team from U.S. Naval Medical Research Unit – Six conducted an epidemiologic survey and environmental assessment, patient interviews, and collected stool samples for analysis in an at risk population of 287 personnel from May 31^st^ to June 3^rd^, 2011. Personnel (n = 241) completed an epidemiological survey (87% response rate) and 67 (27%) reported diarrhea and/or vomiting during the past two weeks. The median duration of illness was reported to be 3 days (IQR 2–4 days) and abdominal pain was reported among 30 (49%) individuals. Presentation to the medical aid station was sought by (62%) individuals and 9 (15%) had to stop or significantly reduce work for at least one day. Microscopy and PCR analysis of 14 stool samples collected from previously symptomatic patients, *Shigella* (7), *Cryptosporidium* (5), and *Cyclospora* (4) were the most prevalent pathogens detected. Consumption of food from on-base local vendors (RR = 4.01, 95% CI = 1.53–10.5, p-value <0.001) and arriving on base within the past two weeks (RR = 2.79, 95% confidence [CI] = 1.35–5.76, p-value = 0.001) were associated with increased risk of developing diarrheal disease. The risk of infectious diarrhea is great among reserve military personnel during two week training exercises. The consumption of local food, prepared without proper monitoring, is a risk factor for deployed personnel developing diarrheal illness. Additional information is needed to better understand disease risks to personnel conducting humanitarian assistance activities in the Latin America Region.

## Introduction

Infectious diarrhea remains a global health problem and a risk to travelers and military personnel deploying to developing regions. Existing epidemiologic data indicates that enterotoxigenic *E. coli* (ETEC), *Campylobacter jejuni*, and *Shigella* spp. (particularly *S. flexneri* and *S. sonnei*) are the most common causes of diarrheal disease among adults and children who live in the developing world as well as among U.S. military personnel deployed to these areas [1], [2], [3].

Diarrheal illness is one of the most common infectious risks among short-term travelers to the developing world, with some studies indicating over 50% of travelers being affected during a two week visit to an endemic country [4], [5]. In a series of 784 American tourists traveling in the developing world for a median 19 days, 46% reported at least one episode of diarrhea (Hill, 2000), while Scottish tourists in Central and South America reported comparable rates of diarrhea (39.5%) [6]. On the other hand, a cohort of 36 Peace Corps volunteers in Guatemala developed 4.7 episodes of diarrhea over a mean 1.8 years of follow-up; 6.1 episodes/person-year occurred in the first 6 months, declining to 3.6 episodes/person-year after 12 months [7].

Among military populations, there were diarrheal disease studies conducted in the Middle East during Operation Bright Star. In 1989, up to 44% of personnel reported diarrheal disease with ETEC (49%) as the predominant pathogen identified [8]. During surveillance activities in 2001, 9.3% of troops reported a diarrheal episode and in 2005, diarrheal disease was prevalent with 35 cases of diarrhea/100 person-months and contributed to 17 non-combat related illnesses/100 person-months [9]. In personnel deploying to Iraq or Afghanistan in 2003 to 2004, 78.6% of troops in Iraq and 54.4% of those in Afghanistan experienced diarrhea, with 80% seeking care from their unit medic; eating local food from non-U.S. sources was associated with an increased risk of illness [10]. Outpatient medical surveillance of U.S. forces during missions conducted in Latin America showed an overall attack rate of 26%, with off-base travel and ice consumption being associated with higher reported disease rates [11]. These studies have demonstrated the risk that diarrheal illness presents to military operations and the risks associated with local food sources.

As part of building partnerships with Latin American nations, the U.S. military historically has embarked on a number of humanitarian operations in the region. Closer interaction with local populations may be necessary in humanitarian operations, increasing potential risks for disease transmission. *Beyond the Horizons* is a U.S. Southern Command sponsored 16-week joint, humanitarian and civic assistance exercise conducted by various U.S. military components scheduled from February to June each year in Latin America. In 2011, activities included engineering, dental, and medical projects to aid citizens in the local vicinity of San Vicente, El Salvador, in areas affected by natural disaster in late 2009. This report describes an outbreak of diarrhea among U.S. military personnel deployed to *Beyond the Horizons* in May 2011.

## Methods

### Study Site and Subjects

Approximately 300 individuals from the 130^th^ Maneuver Enhancement Brigade (MEB) were detached to the Base Poligono in San Vicente, El Salvador as part of a military training and humanitarian assistance mission. The mission took place between February and June 2011, and approximately 75% of the personnel rotated every two weeks. On May 27^th^, 2011, the health unit of the base detected an increase in the number of diarrhea cases presenting for care at the base medical aid station. The U.S. Naval Medical Research Unit Six (NAMRU-6) provided assistance to determine the etiology and mechanism of the outbreak. An outbreak investigation team composed of five personnel was arranged and arrived to El Salvador on May 31st. Working in coordination with the health unit on base, the outbreak team reviewed local records and medical charts, conducted an epidemiologic survey and environmental assessment, patient interviews, and collected stool sample for further laboratory analysis.

### Epidemiologic Surveys

After reviewing records and medical charts, the NAMRU-6 team developed a 61-question survey regarding demographics, health status, clinical symptoms, and food consumption habits during the last two week deployment period (May 21^st^ to June 4^th^, 2011). The survey was administered to all brigade personnel during a scheduled daily informational meeting. The surveys were completed by each individual voluntarily. The case definition for diarrhea was broadened to one or more loose stool episodes (compared to the usual definition of 3 or more loose stools in 24 hrs or at least 2 stools accompanied by fever, blood, etc.) to increase its sensitivity and to capture all possible gastrointestinal illness cases for epidemiologic analysis and targeted stool collection.

An additional survey was administered to three units with the highest prevalence of diarrheal illness to further assess daily food consumption habits during the prior two weeks of the deployment. This survey was developed after discussion with personnel on base about dining options and specific food choices available.

### Environmental Assessment

The environmental assessment conducted on Base Poligono included tent city (living quarters), latrines, hygiene facilities, the potable water distribution system, dining facility (DFAC), on-base vendor locations, and medical aid station. Logistic difficulties prevented the assessment of off-base work sites and food providers. No environmental sampling was conducted. Vendor food sources were not available after May 27^th^, 2011 because on-base vendor facilities and off-base work site food selling practices were stopped following the diarrheal outbreak as a prevention measure. Testing of the base water distribution system and point-of-use locations were performed by the Base personnel in accordance with U.S. Army standards [12] and before the arrival of the NAMRU-6 team.

### Specimen Collection and Laboratory Testing

Stool samples were collected from volunteers in the at-risk population between June 1^st^ and June 3^rd^, 2011. Samples were coded and linked to questionnaires. Fecal smears were made for immediate on-site field microscopy analysis. In addition, sample was preserved in sodium acetate, acetic acid and formalin (SAF) from all volunteers providing a sample, and in potassium dichromate from those volunteers providing a sample with a soft or loose consistency before transportation and further microbiological analysis by microscopy and PCR at NAMRU-6 in Lima, Peru.

### Polymerase Chain Reaction Amplification

Stool samples were tested by PCR for the presence of enteropathogen nucleic acid. PCR testing was conducted for the following virulence markers or bacteria as previously described: (1) ipaH (*Shigella sp*./Enteroinvasive *E. Coli*) [13] (2) *Campylobacter sp.* [14]. PCR testing was conducted as previously described for the following parasites: (1) *Cyclospora cayetanensis* [15], (2) *Cryptosporidium parvum* [16], (3) *Entamoeba histolytica*/*dispar* [17], (4) *Giardia lamblia* [18]. All Amplified PCR products were identified by gel electrophoresis, 1.5% or 2% agarose stained with ethidium bromide and viewed under ultra-violet light using the Bio-Rad Gel Doc XR Universal Hood II.

Real Time RT-PCR testing was conducted to detect norovirus using primers and probes for the polymerase gene of both genotypes I and II (GI and GII), as previously described [19].

### Statistical Analysis

All data was entered into MS Access (Microsoft Inc., Redmond, WA, USA). Data was imported into SAS v9.2 (SAS, Cary, NC), which was used for all statistical analyses. Simple and multiple generalized linear models regression for binomial family and logarithmic link were applied to calculate the relative risk (RR) for factors associated with the cumulative incidence of diarrhea. The Akaike Information Criterion (AIC) was used to determine model fitness. First, we analyzed the data from the first questionnaire applied to all subjects and then we analyzed the results of the second questionnaire applied to only a subset with higher incidence. For the multivariate model using data collected from all personnel, we included the questions that had a statistically significant association with diarrheal illness. All confidence intervals are calculated with 95% confidence and statistical significance was determined at 0.05.

### Ethical Considerations

This activity was deemed an outbreak investigation and determined to not be human subjects research by the Institutional Review Board of NAMRU-6. No informed consent was requested because the activity was conducted fulfilling a required public health mandate. Survey responses and stool samples were voluntary and analyzed de-identified.

## Results

### Diarrheal Illness

**Figure 1:**
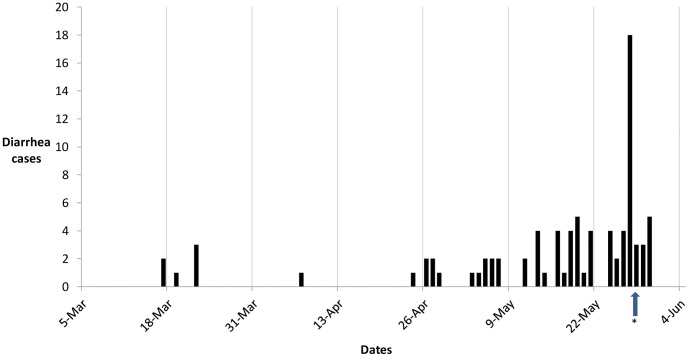
Epidemic curve of personnel reporting to the Medical Aid Station with diarrhea, San Vicente, El Salvador, 2011. The start of each two week deployment periods is indicated by the date. *Indicates the time at which vendors were no longer allowed on base to serve food.

Based on passive surveillance data from the medical aid station, the epidemic curve shows an increase in the number of diarrhea cases starting at the end of April and going through the beginning of June, 2011 (Figure 1). Over this time period, 11, 25, and 39 personnel reported to the medical aid station during the two week rotation periods of April 26^th^ – May 8^th^, May 9^th^ – May 22^nd^, and May 23^rd^ – June 4^th^, respectively. The peak of diarrheal illnesses occurred on May 27^th^, with 18 personnel reporting to the medical aid station. Cases were treated with Ciprofloxacin 500 mg twice daily for three days and metronidazole 500 mg thrice daily for seven days after the diagnosis of *E. histolytica* was made by a local laboratory.

The epidemiological survey had a completion rate of 87% (241/287 personnel) and 67 individuals met the diarrheal outbreak case definition of one or more loose stools in the two-week deployment period, a diarrheal attack rate of 27.8%. Descriptive analyses of the cases revealed that 83.8% (202/241) were male and the median age was 27 years (IQR 22–37) (Table 1). Among patients reporting diarrhea, symptoms included abdominal cramping (49.2%), headache (32.8%), nausea (27.9%), dehydration (23.0%), vomiting (8.2%), fever (8.2%), and one patient reported the presence of blood in their stool. The median duration of illness was 3 days (IQR 2–4 days). Medical care was sought by 62.3% of cases. Among those not seeking care (37.7%), 17.4% self-medicated. Nine personnel with diarrhea (14.8%) reported stopping or significantly reducing work for at least one day. Pre-deployment preventive medicine information was reported to have been received by 35.3% of personnel, with the majority recalling the topics of malaria, personal hygiene and diarrheal illness.

**Table 1:** Demographic and Clinical Characteristics of Study Population.

Clinical and Demographic information	Overall (n = 241)	%
Age - years (median - IQR)	27	IQR 22–37
Gender - male	202	83.8
Have you been in a developing country in the last year?	41	17.1
What country?		
- Latin America/Caribbean	23	55.2
- Middle East	17	40.8
- Other	1	2.4
Diarrhea or vomiting in the last two weeks?	67	27.8
Duration of diarrhea or vomiting? (days)	3	IQR 2–4
Fever	5	8.2
Blood in Stool	1	1.0
Abdominal Pain	30	49.2
Dehydration	14	23.0
Nausea	17	27.9
Headache	20	32.8
Present at sick call due to diarrhea or vomiting?	38	62.3
Stop or signifcantly reduce work at least one day?	9	14.8
how many days miss work?		
- one day	5	55.5
- two days	4	44.4
Receive IV fluids?	4	6.6
Receive antibiotics?	35	57.4
Antibiotic self-medication?	4	6.6
Preventive Medicine Information before Deployment	85	35.3
Method of Delivery		
- paper-based	25	29.1
- Lecture	39	45.4
- online training	6	6.9
- other	7	8.1
Topics covered in training		
- Malaria	64	74.4
- dengue	33	38.3
- diarrhea	51	59.3
- injuries	40	46.5
- healthcare access	33	38.4
- insurance	11	12.8
- personal hygiene	62	72.1
- other	7	8.1

The bivariate analysis of the completed surveys indicates that the individuals arriving on base within the past two weeks were at higher risk for developing diarrheal disease (estimated risk ratio [RR] = 2.79, 95% confidence [CI] = 1.35–5.76, p-value = .001) (Table 2). In addition, the consumption of meals from on-base local vendors was statistically associated with illness (RR = 4.01, 95% CI = 1.53–10.5, p-value <.001) (Table 2). Consumption of food off base that was cooked (RR = 0.35, 95% CI = 0.22–0.54, p-value = .005) or served hot (RR = 0.41, 95% CI = 0.25–0.67, p-value = .007) was considered to be protective from diarrheal disease. Other factors such as adding hot sauces to off-base foods, eating at street vendors off base and drinking non-bottled beverages off-base were not associated with risk of diarrhea. Multiple regression analysis using the significant variables from bivariate analysis showed that only arriving on base within the past two weeks (RR = 2.85, 95% confidence CI = 1.31–6.18, p-value = .008) and eating at local vendors on base (RR = 3.91, 95% confidence CI = 1.59–10.18, p-value = .005) were significantly associated with diarrheal risk (Table 2).

**Table 2:** Association of risk factors with acquisition of illness among all survey takers (n = 241) and multiple regression analysis of risk factors for acquisition of diarrheal illness.

			Univariate Analysis		Multivariate Analysis		
Question	Yes/No	Diarrhea	Risk Ratio	p-value	Risk Ratio	95% CL	p-value
Arriving on base within past two weeks	Yes	60 (33.7%)	2.79	0.001	2.85	1.31–6.18	0.008
	No	7 (12.1%)					
Eat off base in last two weeks?	Yes	50 (31.8%)	1.51	0.077			
	No	17 (20.9%)					
Were off base foods cooked?	Yes	43 (29.2%)	0.35	0.005*			
	No	5 (83.3%)					
Were off base foods served hot?	Yes	42 (29.1%)	0.41	0.007*			
	No	7 (70.0%)					
Did you add hot sauces to off-base foods?	Yes	11 (23.4%)	0.67	0.157			
	No	37 (34.9%)					
Eat at street vendors off base in last two weeks?	Yes	28 (28.2%)	1.01	0.938			
	No	37 (27.8%)					
Eat at local vendors (non-DFAC) on-base?	Yes	59 (32.7%)	4.01	<0.001	3.91	1.50–10.18	0.0051
	No	4 (8.1%)					
Drink non-bottled beverages off-base in last two weeks?	Yes	15 (37.5%)	1.41	0.157			
	No	51 (26.4%)					

Two units (n = 80) accounted for more than half of the cases of diarrhea (45%, 36/80). The detailed survey among this group indicated that separate consumption of either tacos or papusas on May 23^rd^ or May 25^th^ from vendors on base were statistically associated with a higher incidence of diarrheal illness (Table 3). Individuals who ate neither tacos on May 23^rd^ nor papusas on the 25^th^, reported lower than average diarrheal rates (10%, 4/39). Among personnel who only ate either tacos on May 23^rd^ or papusas on the 25^th^, 80% (12/15) and 76% (16/21) reported diarrheal illness, respectively. Personnel who ate both tacos on May 23^rd^ and papusas on May 25^th^, 80% (4/5) reported diarrheal illness. Overall, 63.8% (23/36) of personnel from these two units who developed diarrheal illness ate either tacos on May 23^rd^ or papusas on May 25^th^. A similar but less striking pattern was observed analyzing in detail eating tacos on the 25^th^ or papusas on the 23^rd^, suggesting that reporting of these foods may be mainly correlated with the actual vehicles of infection but not necessarily a causative factor.

**Table 3:** Food Consumption among three units with highest incidence of diarrheal disease (n = 80).

			Univariate Analysis	
Question	Yes/No	Diarrhea	Risk Ratio	p-value
Ate Tacos for dinner onthe 23^rd^?	Yes	12 (33.3%)	2.13	0.0029
	No	3 (6.9%)		
Ate Tacos for dinner onthe 25^th^?	Yes	14 (37.8%)	2.09	0.0023
	No	4 (9.3%)		
Ate Papusas for dinner onthe 23^rd^?	Yes	14 (38.9%)	1.87	0.01
	No	6 (13.9%)		
Ate Papusas for dinner onthe 25^th^?	Yes	16 (43.2%)	2.14	0.0014
	No	5 (11.6%)		

### Laboratory Results

Fifty-one subjects provided stool samples and twelve with a soft or loose consistency were analyzed on-site by field microscopy for parasites. *Cyclospora* spp. was identified in one symptomatic patient receiving ciprofloxacin and metronidazole. In consultation with the senior medical officer, this patient’s treatment regimen was changed to include an antiparasitic agent with effectiveness against *Cyclospora.*

PCR testing was performed on 14 stool samples from patients who had presented with a soft or loose stool and had reported diarrhea over the past two weeks. PCR analysis identified pathogens in 78% (11/14) of samples tested with 43% (6/14) testing positive for multiple pathogens. The majority of samples (7/14) were positive for a *Shigella* spp. or enteroinvasive *E. coli* virulence marker (ipaH) (Table 4).

**Table 4:** PCR detection of enteropathogens (n = 14) among 14 samples collected from personnel reporting diarrhea in El Salvador.

Sample	*Cyclospora cayetanensis*	*Cryptosporidium*	*Entamoeba spp.*	*Giardia lamblia*	norovirus	IpaH	Campylobacter
1	**Positive**	Negative	Negative	Negative	Negative	**Positive**	**Positive - ** ***C. jejuni***
2	**Positive**	Negative	Negative	**Positive**	Negative	**Positive**	Negative
3	Negative	Negative	Negative	Negative	Negative	**Positive**	Negative
4	**Positive**	**Positive**	Negative	Negative	Negative	Negative	Negative
5	**Positive**	Negative	Negative	**Positive**	Negative	**Positive**	Negative
6	Negative	Negative	**Positive**	Negative	Negative	Negative	Negative
7	**Positive**	**Positive**	Negative	Negative	Negative	Negative	Negative
8	Negative	**Positive**	Negative	Negative	Negative	**Positive**	Negative
9	Negative	**Positive**	Negative	Negative	Negative	Negative	Negative
10	Negative	Negative	Negative	Negative	Negative	Negative	Negative
11	Negative	Negative	Negative	Negative	Negative	Negative	Negative
12	Negative	Negative	Negative	Negative	**Positive**	Negative	Negative
13	Negative	Negative	Negative	Negative	Negative	**Positive**	Negative
14	Negative	Negative	Negative	Negative	Negative	**Positive**	Negative
Total	5 (35.7%)	4 (28.6%)	1 (7.1%)	2 (14.3%)	1 (7.1%)	7 (50.0%)	1 (7.1%)

The original sample from which *E. histolytica* had been identified by a local laboratory was not available for further analysis. Testing of a subsequent stool sample of the same case collected after treatment with metronidazole had begun was negative for *E. histolytica* by microscopy and PCR analysis.

### Food and Beverage Assessment

Food and beverage sources for U.S. personnel were available from the on-base Dining Facility (DFAC), Meals-Ready-To-Eat (MREs), and local vendors both on and off-base. The DFAC prepared three meals daily and was established on base in accordance with Army regulations [20]. Meals were standard prepackaged food approved by the U.S. Army, heated on site, opened, and served directly to service members. Disposable plates and utensils were utilized and waste from all food products was disposed daily.

The only option for breakfast was either MRE or DFAC. Food options for lunch and dinner included DFAC, MRE, or local vendors. There were two food vendors on base, named “upper” and “lower” vendors, that prepared meals daily from fresh food purchased locally and brought to base daily by local personnel. Local vendors were not contracted nor assessed for food safety practices prior to providing services. Off-base vendors were local street sellers who took lunch orders from U.S. personnel at work-sites and prepared meals in personal home or local restaurant kitchens.

Observation and assessment of vendor food preparation practices could not be completed because vendors were closed as a mitigation strategy for this diarrheal outbreak. Key informant interviews reported on-base vendor food was brought to base raw or uncooked in buckets, left unrefrigerated outside, and prepared under outdoor open, covered structures on grills and wood tables. The same cooking utensils were used to prepare raw food and serve cooked meat products and vegetables. Meats were reported to not be cooked completely prior to serving and many raw vegetables were served without knowledge of cleaning from potable water sources. Off-base work site vendors brought cooked meals to U.S. personnel presumably prepared in local homes or restaurants.

### Potable Water Assessment

On-base water was provided by the primary local city source. The base Reverse Osmosis Water Purification Unit (ROWPU) was not functional. Water was stored in a 10,000 gallon water storage bladder (blivits) and made potable by chlorination in accordance with U.S. Army standards [12]. Potable water was distributed by soft hose and made available for consumption at two water tanks. This potable water was served at multiple cooler distribution points in the DFAC. Local bottled water was also available for purchase on base. After identification of this diarrheal outbreak, both water tanks were drained, shock chlorinated, and re-filled, and additional multiple water quality checks were conducted within the base water distribution system.

### Sanitation Assessment

Base latrines were contracted Portalet-type facilities that were drained and cleaned daily. Each latrine had a hand-washing sink with water foot pump and soap. There were two hand-washing stations at the entrance to the DFAC. After identification of this diarrhea outbreak, each individual latrine hand washing sink and DFAC hand-washing stations were routinely chlorinated. In addition, an individual was assigned to the DFAC entrance to ensure hand washing by all personnel during meal hours. Personal hygiene facilities were cleaned daily.

## Discussion

This study describes an outbreak of diarrheal illness among deployed U.S. military personnel during Operation *Beyond the Horizon* in El Salvador. We identified an increasing trend of diarrheal illness occurring throughout the course of the operation and an association with the consumption of food sold from local vendors on-base. Diarrheal illness is a risk to deployed U.S. military units and travelers throughout the world. In the humanitarian and civic assistance mission setting, operating off base, working in local communities, and sharing meals are important cultural components that present infectious disease risks to deployed personnel [8], [10].

The burden of diarrheal illness represented by the epidemiologic curve highlights a recurring increase in diarrheal cases during each two-week deployment period, especially during the last three deployment periods. This data is representative of other studies showing diarrheal illness being a common burden of illness in short-term deployed U.S. military units [21], [22], [23], [24]. The shorter duration of this deployment and inability to follow-up with personnel that had already completed their two week rotation may, in part, explain the lower prevalence of diarrheal illness compared to studies in Iraq or Afghanistan. However, there have been few studies on the epidemiology of diarrheal illness and the effectiveness of preventive strategies in deployed military units in Central and South America [11], [25], [26].

With the majority of personnel for these operations rotating every two weeks, it is critical to ensure that public health measures are maintained throughout the duration of the operation. While situations such as local food sources may not be covered under governing regulations, preventive medicine principles should be applied to the greatest extent possible according to the situation (e.g. training and monitoring of local vendors). In addition, part of maintaining appropriate public health measures includes preventive medicine education, which the majority of personnel report they did not receive. Ensuring the continuity of public health principles can help prevent diarrheal disease.

Epidemiologic and statistical analysis of food consumption histories obtained from surveys suggests that the consumption of food from local vendors, specifically tacos and papusas, on base was most significantly associated with illness. These findings are consistent with environmental evidence indicating that improper cooking procedures at local vendors could have resulted in the growth of microbiological organisms. However, vendors were not inspected nor trained on proper food handling practices prior to operating on base, and these hypotheses could not be confirmed in our investigation.

An important feature of stopping this diarrheal outbreak was the prompt recognition of increasing cases by U.S. military medical personnel and the implementation of mitigation strategies that decreased incidence before the arrival of the NAMRU-6 team. The banning of local vendor food is an important prevention strategy for diarrheal disease outbreaks; however, the availability of alternative food options during deployment is important for troop morale and building partnerships. The offering of local food by host nation people is a sign of hospitality. Under such circumstances, the refusal of food offerings could be perceived as rude and counter to the U.S. diplomatic and training goals. However, diarrheal illness among U.S. military personnel caused on average the loss of 1.5 duty days and 27% of personnel were affected during a 2-week deployment period. Such impact can affect mission readiness and the ability to complete projects on time. As part of the efforts to promote readiness and shared cultural experiences, proper food handling and hygiene instruction should be conducted for vendors that are coming on base to serve foods and at off-base locations providing meals to military personnel in the field.

The laboratory results identified numerous bacterial and parasitic pathogens present in the stool samples from individuals that had reported diarrheal illness. The illness observed in this outbreak was characterized primarily by diarrhea and abdominal pain, with very few cases of vomiting or fever. These clinical features closely resembled those of a bacterial etiology, and the predominant pathogen identified was for ipaH, present in all four *Shigella* spp. and enteroinvasive *E. coli*. An extensive series of laboratory tests for bacteria, viruses and parasites was performed on samples from this outbreak investigation. The polymicrobial findings of the stools that were tested suggest that heavy contamination of vendor prepared food from fecal material may have played a role in this outbreak. The use of molecular methods may represent an increased sensitivity in the detection of pathogens compared to conventional culture and microscopy based methods [27]. We were unable to perform bacterial culture on the remaining sample and microscopic examination could confirm only one of the *Cyclospora* positive samples. However, given the inability to collect control samples, the identified etiologies should be cautiously interpreted in the context of this outbreak investigation. In addition, we were not able to assay for the most common cause of deployment-associated diarrhea, diarrheagenic *E. coli* (e.g. enterotoxigenic *E. coli*, enteroaggregative *E. coli*). The limitations in pathogen identification in this type of setting highlight the need for improved field diagnostics.

Previous studies have also demonstrated the importance of bacterial etiologies in diarrheal disease among deployed military units [1], [8], [10], [21], [28]. Due to the potential of diarrheal illness to disrupt military missions, the U.S. military has placed a high priority on the development of effective vaccines and other prophylactic measures against the most common enteropathogens like ETEC, *Campylobacter* and *Shigella* species [29], [30], [31], [32]. Short-term travelers and deployed military units may serve as important populations for testing preventive strategies.

This outbreak investigation in El Salvador highlights the need for more information on infectious disease risks to deployed military personnel in Latin America to help guide prevention measures and empiric treatment when routine laboratory or diagnostic support is not available. Information gathered from infectious disease surveillance activities during these operations will help prepare military personnel and travelers visiting the region.
